# Quality of life in nursing home residents with pain: pain interference, depression and multiple pain-related diseases as important determinants

**DOI:** 10.1007/s11136-019-02290-x

**Published:** 2019-09-21

**Authors:** A. Brandauer, S. Berger, N. Freywald, I. Gnass, J. Osterbrink, D. Seidenspinner, P. Kutschar

**Affiliations:** 1 Institute of Nursing Science and Practice, Paracelsus Medical University, Strubergasse 21, 5020 Salzburg, Austria; 2 Campus Großhadern, Hospital of the Ludwig-Maximilians-University, Marchioninistraße 15, 81377 Munich, Germany

**Keywords:** HRQOL, Quality of life, EQ-5D-3L, Nursing home, Dementia, Pain

## Abstract

**Aim:**

Quality of life is an essential outcome parameter in geriatric research and presents an important indicator for the evaluation of care treatments. The present study analyses potential impact factors on health-related quality of life (HRQOL) of nursing home residents (NHR) who are in pain.

**Methods:**

Data came from the cRCT ‘PIASMA’. Statistical analyses of 146 respondents were carried out by multiple linear regressions based on the EQ-5D index (Euroquol Quality of Life) as dependent variable. Potential impact factors were applied and categorised in five blocks: pain intensity and interference (according to the Brief Pain Inventory), intervention effect, sex and age, pain-related diagnoses, and scales regarding depressive symptoms and cognitive impairment (based on the Geriatric Depression Scale and the Mini-Mental State Examination).

**Results:**

On average, residents showed a pain intensity of 18.49, a pain interference of 29.61, a MMSE score of 22.84, a GDS score of 5.65 and an EQ-5D index of 0.52. Residents with more diagnoses, more depressive symptoms, and a higher pain interference showed a significantly reduced HRQOL.

**Conclusion:**

Findings underline the importance of identifying and applying treatment options for both pain (especially interference) and depressive disorders to maintain HRQOL of NHR.

## Introduction

Quality of life has become an essential issue in geriatric research and an important indicator for the evaluation of care treatments and healthcare policies [1–3]. Many previous studies have shown that pain is a relevant factor influencing health-related quality of life (HRQOL). Pain affects all aspects of daily life (e.g. quality of sleep, mobility) and therefore, most domains of HRQOL. Extent, duration, acuteness, intensity, affectivity, and meaning of pain determine type and strength of the effect of pain on HRQOL [2, 4–6]. Pain is a central problem in the geriatric population, especially in nursing home residents (NHR) [5–7]. Next to pain, NHR frequently have multiple diseases and depressive disorders, which are also known to lower HRQOL [1, 7, 8]. The empirical evidence regarding the relationship of pain and HRQOL of NHR is still limited especially when considering other co-determinants [3, 5, 7]. Therefore, further research and a broader understanding of the multidimensional impact of pain on HRQOL of NHR is needed to provide indications for target-group-specific treatment and finally, maintain, restore or improve the HRQOL of NHR [3, 5, 7]. The present study addresses this issue and adds further evidence on influencing factors concerning HRQOL of NHR who are in pain. Moreover, analyses include the rarely addressed, but important factor ‘pain interference’ [4, 7].

## Methods

### Data and procedures

**Fig. 1:**
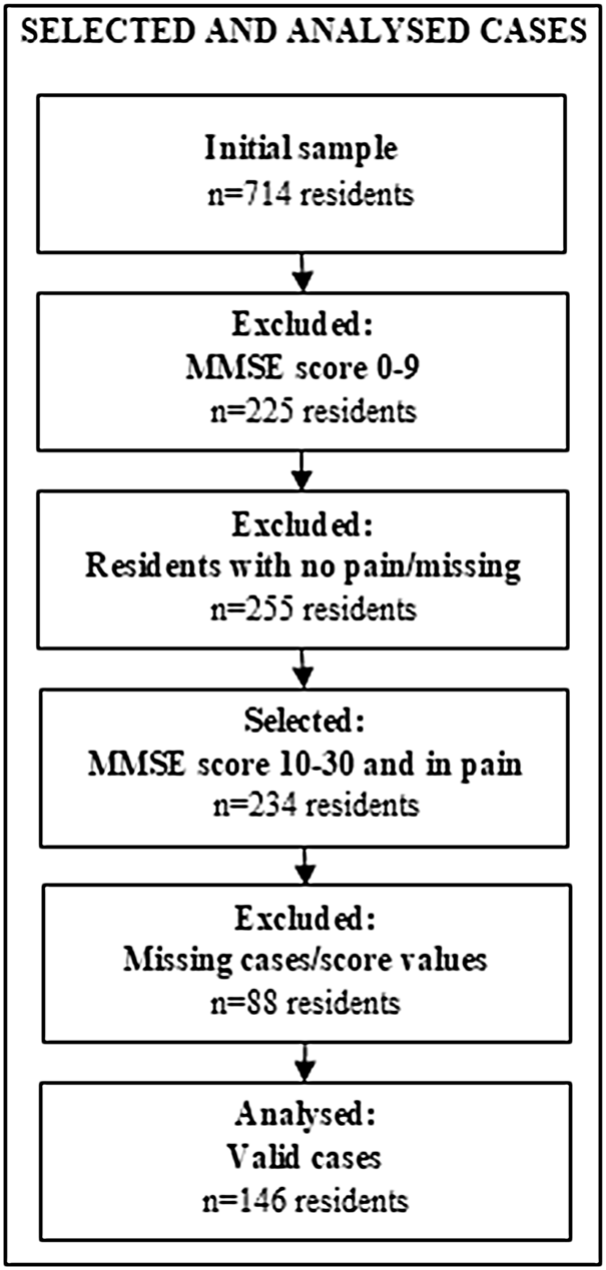
Included cases

Data of 146 residents came from the cluster-randomised controlled trial (cRCT) ‘PIASMA’ (2016–2018) [9], which included 15 randomly selected nursing homes in Bavaria, Germany. The intervention comprised an educational package in pain management for nurses (i.e. education of pain nurses and pain care assistants, web-based training and quality circles). The inclusion criteria for NHR were: Being at least 60 years old, living permanently in the facility, and giving informed consent to participate. Residents with limited German language skills, living with congenital multiple disabilities, acute diseases or finding themselves in life-threatening situations were excluded. Residents were initially screened with the Mini-Mental State Examination (MMSE, German). The number of correct answers were transferred into a summary score, which ranged from 0 [maximum cognitive impairment (CI)] to 30 points (no CI) [10]. NHR were stratified into two groups of CI: NHR able to perform self-assessment (no/mild or moderate CI; MMSE score: 10–30) and NHR not able to perform self-assessment (severe CI; MMSE score: 0–9) [11]. The present study focused on residents who were able to perform self-assessment^1^ and reported to be in pain at the moment of survey. Details on selected and analysed cases are shown in Fig. 1.

### Measures

The EQ-5D-3L Questionnaire (German) was applied to measure the HRQOL. Next to a visual analogue scale (VAS), which presents the respondents’ self-rated health state (from 0 = ‘worst imaginable health state’ to 100 = ‘best imaginable health state’), the EQ-5D-3L comprises five dimensions with three levels each (no, some, extreme problems). To compute a single summary index (EQ index) with the endpoints ‘worst imaginable health state’ (= 0.02) and ‘best imaginable health state’ (= 1) the visual analogue scale valuation technique was used [12–14]. Although the EQ-5D-3L was not specifically constructed for people with CI, several studies proved its applicability for people with dementia and institutionalised older adults [15–18].

The validated Brief Pain Inventory (BPI, German) was used to measure pain [19]. The BPI assesses pain in terms of intensity and interference. Pain intensity was computed as a summary index out of four items (worst, least, average, current pain), each rated on the numeric rating scale (0 = no pain; 10 = pain as bad as you can imagine). The pain interference summary index represents seven items regarding interference on daily activities due to pain on a ten-point rating scale (0 = no interference; 10 = interferes completely).

Depressive symptoms were assessed by the Geriatric Depression Scale (GDS, German) [20]. GDS consists of 15 dichotomous items and measures symptoms for depression during the last seven days. GDS results in a score between 0 and 15 points, whereby a higher score indicates more relevant signs of depression and a cut-off > 5 points was interpreted as non-ignorable [21].

Gender, age, and pain-related diagnoses were extracted from nursing documentation. Next to the specific pain-related diagnoses (i.e. neuropathy, tumour, musculoskeletal disorders, chronic wounds and diabetes), each resident’s number of documented diagnoses was computed.

### Analyses

Statistical analyses were carried out using IBM SPSS 24.0 applying multiple linear regressions [Ordinary Least Squares (OLS)] to predict the EQ index. Independent variables were generated within two models: The first model (basic model) included variables, which had been identified as relevant impact factors for HRQOL in previous studies (find references for each factor in Table 1). The indicators were structured hierarchically into five blocks (Table 1), whereby variables of each block were added simultaneously.

**Table 1:** Included predictors per block in OLS-regressions

Block	Predictors and rationale
1	Pain (interference and intensity) as main predictor [2, 4, 5]
2	Intervention effect: Since the PIASMA-study was a cRCT, analyses were adjusted for the intervention effect (time × group interaction)
3	Age and gender* are frequently discussed as predictors for HRQOL [2, 3]
4	Pain-related diagnoses*: Many studies declare that chronic diseases and comorbidities influence HRQOL [1, 7, 8]. Hence, confirmed diagnoses (i.e. neuropathy, tumour, musculoskeletal disorders, chronic wounds, diabetes) were included
5	Depressive symptoms and cognitive impairment are phenomena known to affect HRQOL [1, 3, 5, 7, 22, 23]

*Extracted from nursing documentation, according to current medical definitions (ICD)

*OLS* ordinary least squares, *cRCT* cluster-randomised controlled trial; *HRQOL* health-related quality of life

The second model (final model) referred to the results of the first model. Indicators were selected based on effect size (*β*), *p* value, and collinearity statistics [i.e. variance inflation factor (VIF), tolerance and condition index] of each factor and the adjusted *R*^2^ of the overall model.

## Results

### Sample

In total, 146 residents were analysed (Fig. 1). The average age was 82 years, and one-third was male. 42% of the participants had musculoskeletal disorders, approximately a fifth had a tumour, whereas 12%, 13% and 15% had neuropathy, diabetes and chronic wounds, respectively. The average pain intensity was 18.49 (indicating ‘moderate pain’), and the mean pain interference index was 29.61. On average, respondents had an MMSE of 22.84 (indicating ‘mild CI’). The mean GDS score was 5.65 (indication ‘mild-to-moderate depression’). Most residents had problems with pain/discomfort and mobility (97.2% and 83.5%, respectively), the fewest with anxiety/depression (45.2%). The average EQ VAS was 55.13 and the mean EQ index 0.52. Details are shown in Table 2.

**Table 2:** Characteristics of included respondents

Participant characteristics and predictors	
Age mean ± SD (*n*)	81.55 ± 9.74 (146)
Gender male %_valid_ (*n*)	34.9 (51)
Diagnoses (multiple response) %_of cases_ (*n*)	
Musculoskeletal disorders	41.8 (61)
Tumour	18.5 (27)
Chronic wounds	15.1 (22)
Diabetes	13.0 (19)
Neuropathy	11.6 (17)
Pain intensity index [0–40] mean ± SD (*n*)	18.49 ± 7.23 (146)
Pain interference index [0–70] mean ± SD (*n*)	29.61 ± 16.02 (146)
MMSE score [10–30] mean ± SD (*n*)	22.84 ± 4.90 (146)
GDS score [0–15] mean ± SD (*n*)	5.65 ± 3.66 (146)

Outcome EQ-5D-3L ‘quality of life’			
EQ descriptive system (*n*)	No problems	Some problems	Extreme problems
Mobility %_valid_ (146)	16.4	80.1	3.4
Self-care %_valid_ (146)	39.0	48.6	12.3
Usual activities %_valid_ (146)	31.5	50.7	17.8
Pain/discomfort %_valid_ (146)	2.7	75.3	21.9
Anxiety/depression %_valid_ (146)	54.8	40.4	4.8
EQ index [.02–1] mean ± SD (*n*)			.52 ± .24 (146)
EQ VAS [0–100] mean ± SD (*n*)			55.13 ± 21.51 (134)

*SD *= standard deviation, *n *= sample size, *MMSE *= Mini-Mental State Examination, *GDS *= Geriatric Depression Scale, *EQ *= EQ-5D-3L (Euroquol Quality of Life), *VAS *= visual analogue scale

### OLS models

Predictors listed in Table 1 were blockwise and hierarchically selected in five steps. A comparison of all steps of the basic model showed that the adjusted *R*^2^ of the fifth step was significantly better than that of the other steps (*F* change statistics < 0.001). Hereafter, assumptions for OLS regression analysis including casewise diagnostics, linearity, homoscedasticity, independence, and normality of residuals of the last step of the basic model were tested. According to these tests, the last step of the basic model fulfilled all assumptions except for the boundaries for leverage and condition index. The objective then was to find a reliable model with a condition index below 30.0 and without influential outlying values of the independent variables (Table 3).

**Table 3:** OLS models: predictors of quality of life (EQ index)

		Basic model			Final model		
		Tolerance	VIF	*β*	Tolerance	VIF	*β*
1	Constant (B)	.790			.703		
	Pain intensity index (0–40)	.677	1.476	− .196*	.564	1.772	.005
	Pain interference index (0–70)	.677	1.476	− .334***	.564	1.772	− .446***
2	Constant (B)	.774			.681		
	Pain intensity index (0–40)	.674	1.484	− .210*	.563	1.777	− .010
	Pain interference index (0–70)	.677	1.476	− .334***	.564	1.772	− .445***
	Intervention group (0/1)	.992	1.008	.163*	.996	1.004	.210*
3	Constant (B)	.557			–		
	Pain intensity index (0–40)	.674	1.484	− .211*	–	–	–
	Pain interference index (0–70)	.639	1.566	− .294**	–	–	–
	Intervention group (0/1)	.977	1.024	.161*	–	–	–
	Age (60–105)	.846	1.182	.084	Age and gender had no significant impact		
	Male (0/1)	.877	1.140	.167*			
4	Constant (B)	.604			.739		
	Pain intensity index (0–40)	.644	1.552	− .175*	.555	1.800	− .031
	Pain interference index (0–70)	.575	1.738	− .325**	.564	1.774	− .451***
	Intervention group (0/1)	.923	1.083	.158*	.954	1.048	.181*
	Age (60–105)	.790	1.266	.064	–	–	–
	Male (0/1)	.802	1.246	.141^a^	–	–	–
	Neuropathy (0/1)	.534	1.871	.307**	Deleted because of missing correlation		
	Tumour (0/1)	.751	1.332	.101	–	–	–
	Musculoskeletal disorders (0/1)	.463	2.161	.160	–	–	–
	Chronic wounds (0/1)	.663	1.508	− .057	–	–	–
	Diabetes (0/1)	.927	1.079	.008	–	–	–
	Number of diagnoses	.255	3.924	− .260^a^	.924	1.082	− .144
5	Constant (B)	.893			**.814**		
	Pain intensity index (0–40)	.632	1.582	− .174*	**.552**	**1.812**	− **.064**
	Pain interference index (0–70)	.551	1.814	− .260**	**.476**	**2.101**	− .274*
	Intervention group (0/1)	.914	1.094	.145*	**.954**	**1.049**	.173*
	Age (60–105)	.751	1.332	.013	–	–	–
	Male (0/1)	.795	1.257	.117	–	–	–
	Neuropathy (0/1)	.531	1.884	.309**	Deleted because of missing correlation		
	Tumour (0/1)	.713	1.403	.088	–	–	–
	Musculoskeletal disorders (0/1)	.430	2.326	.134	–	–	–
	Chronic wounds (0/1)	.660	1.515	− .039	–	–	–
	Diabetes (0/1)	.915	1.093	.035	–	–	–
	Number of diagnoses	.242	4.137	− .281*	**.918**	**1.089**	− .171*
	MMSE score (0–30)	.875	1.143	− .095	–	–	–
	GDS score (0–15)	.812	1.231	− .246**	**.785**	**1.273**	− .348***
	Condition index	45.733			**11.323**		
	Adjusted *R*^2^	0.344^b^			**0.324**^**c**^		
	Durbin-Watson statistic	1.635			**2.133**		
	Studentized residual	All cases between − .3.0 and 3.0			**All cases between − 3.0 and 3.0**		
	Centered leverage value	3 cases > .178			**All cases < .090**		
	Cook’s distance	All cases < 1.0			**All cases < 1.0**		
	Homoscedastic	Approved via scatterplot			**Approved via scatterplot**		
	Normally distributed	Approved via KS-test (*p* > .05)			**Approved via KS-test (*p* > .05)**		
	Linearity	Approved via scatterplot			**Approved via scatterplot**		

*OLS* ordinary least square, *EQ* EQ-5D-3L (Euroquol Quality of Life), *VIF* variance inflation factor, *β* beta coefficient, *B* intercept, *MMSE* Mini-Mental State Examination, *GDS* Geriatric Depression Scale, *R*^*2*^ coefficient of determination, *KS*-*test* Kolmogorow–Smirnow test, *bold* very final model - > used for interpretation

*p value < 0.05; **p value < 0.01; ***p value < 0.001

^a^*p* value < 0.10

^b^*F* change statistics < 0.001

^c^*F* change statistics < 0.001

**Fig. 2:**
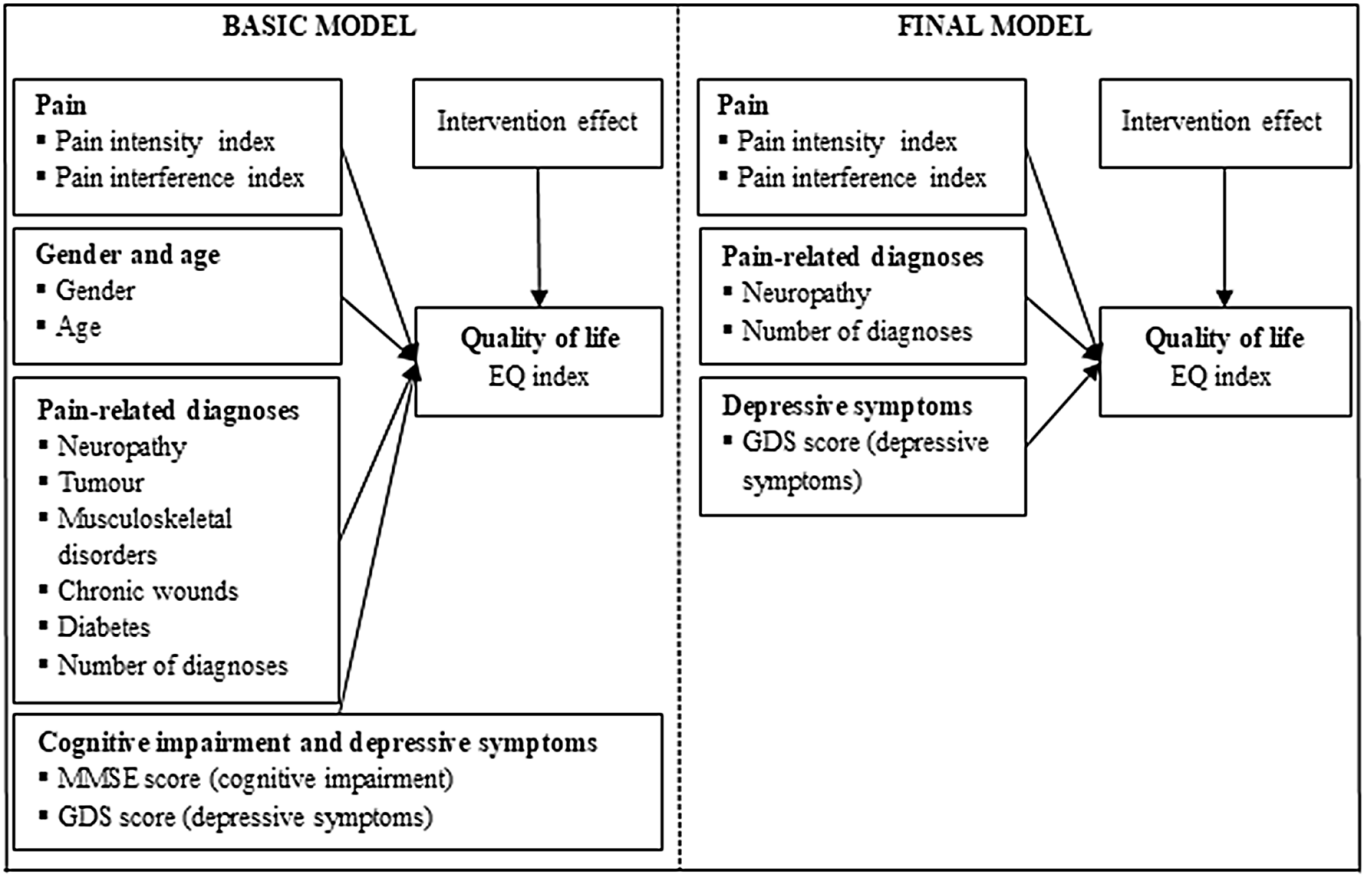
Basic and final model of included predictors of quality of life (EQ index). *EQ* EQ-5D-3L (Euroquol Quality of Life)*; MMSE* Mini-Mental State Examination; *GDS* Geriatric Depression Scale

Therefore, six predictor variables (i.e. pain intensity, pain interference, intervention effect, neuropathy, number of diagnoses and depressive symptoms) were included into the final regression model. Once again, the last step was significantly better at predicting the outcome variable than the other steps (*F* change < 0.001). Analyses showed that main assumptions were not violated but casewise diagnostics showed influential data points that deviated from leverage boundaries. Elimination of these cases led to the very final model (last step of the final model) (Fig. 2; Table 3, bold).

The very final regression model (Table 3, bold) showed significant predictors for HRQOL in NHR and explained 32.4% of variance, which represents a substantial part of the explained variation according to Cohen [24]. Whereas pain interference affected HRQOL in all models, pain intensity showed no significant influence. After adjusting for the intervention effect, the impact of pain interference was reduced minimally (step 2). Adding ‘number of diagnoses’ (step 4) increased the influence of pain interference. Although pain interference remained a significant predictor for HRQOL, adding ‘depressive symptoms’ in step 5 decreased the influence of pain interference. Therefore, residents with more diagnoses (*β* = − 0.171, *p* < 0.05), more depressive symptoms (*β* = − 0.348, *p* < 0.001), and a higher pain interference (*β* = − 0.274, *p* < 0.05) showed a significantly lower HRQOL. Moreover, the implemented educational intervention had a positive impact on HRQOL of the NHR (*β* = 0.173, *p* < 0.05).

## Discussion

Findings show that pain interference, depressive symptoms and number of pain-related diagnoses significantly decrease HRQOL of NHR. These findings converge with the results of previous studies and constitute further evidence that pain is of great significance for HRQOL [1–5, 8, 22, 23]. Regarding pain-related items, it is apparent for both research and practice that interference seems to be more important for HRQOL than intensity. Moreover, single-specific pain-related diagnoses have no significant influence on HRQOL after adding further independent variables, whereas presence of multiple pain-related diagnoses lowers HRQOL of residents significantly. Healthcare professionals should, therefore, pay special attention to individuals with multiple pain-related diseases. The significant influence of depressive symptoms on HRQOL supports previously stated claims to consider psychological aspects, since depression is often comorbid with pain [6, 7]. An adequate multidisciplinary treatment of pain, but also of depressive disorders seems to be essential to maintain or improve HRQOL of NHR. Since a significant intervention effect on HRQOL was observed, nursing-related education concerning pain management should explicitly be considered in further geriatric care. Moreover, previous studies showed that HRQOL can be conceptualized as a psycho-social construct [8, 25]. Further determinants, e.g. relations to family or friends, may have increased the model’s explained variance. Additional potential impact factors like polypharmacy and other diagnoses [23] should also be examined in future research.

The authorized ethics committee (Ludwig-Maximilian University of Munich, Germany) approved the study protocol and the consent procedure of the PIASMA-study (WHO-UTN: U1111-1187-3174). All participants provided informed consent and could withdraw from the study at any time on request.

## Footnotes

1 Separate analyses of the two different MMSE-groups (MMSE score 10–18 and MMSE score 19–30) were not possible due to the small sample size of the group MMSE score 10–18.

## References

[1] León-Salas B, Ayala A, Blaya-Novakova V, Avila-Villanueva M, Rodriguez-Blázquez C, Rojo-Pérez F (2015). Quality of life across three groups of older adults differing in cognitive status and place of residence. Geriatrics & Gerontology International.

[2] Niv D, Kreitler S (2001). Pain and quality of life. Pain Practice.

[3] Beerens HC, Zwakhalen SM, Verbeek H, Ruwaard D, Hamers JP (2013). Factors associated with quality of life of people with dementia in long-term care facilities: A systematic review. International Journal of Nursing Studies.

[4] Müller-Schwefe GHH, Überall MA (2011). Schmerz und Lebensqualität [Pain and Quality of Life]. Gesundh ökon Qual manag.

[5] Rostad HM, Puts MTE, Cvancarova Smastuen M, Grov EK, Utne I, Halvorsrud L (2017). Associations between pain and quality of life in severe dementia: A Norwegian cross-sectional study. Dementia and Geriatric Cognitive Disorders Extra.

[6] Morete MC, Solano JPC, Boff MS, Filho WJ, Ashmawi HA (2018). Resilience, depression, and quality of life in elderly individuals with chronic pain followed up in an outpatient clinic in the city of Sao Paulo, Brazil. Journal of Pain Research.

[7] Jakobsson U, Klevsgård R, Westergren A, Hallberg IR (2003). Old people in pain: A comparative study. Journal of Pain and Symptom Management.

[8] Orfila F, Ferrer M, Lamarca R, Tebe C, Domingo-Salvany A, Alonso J (2006). Gender differences in health-related quality of life among the elderly: The role of objective functional capacity and chronic conditions. Social Science and Medicine.

[9] Kutschar P, Brandauer A, Berger S, Gnass I, Seidenspinner D, Freywald N (2017). PIASMA—Projekt zur Implementierung eines adäquaten Schmerzmanagements in der Altenhilfe. Schmerznachrichten.

[10] Folstein MF, Folstein SE, Fanjiang G (2001). MMSE Mini-Mental State Examination clinical guide.

[11] Lukas A, Niederecker T, Gunther I, Mayer B, Nikolaus T (2013). Self- and proxy report for the assessment of pain in patients with and without cognitive impairment: Experiences gained in a geriatric hospital. Zeitschrift fur Gerontologie und Geriatrie.

[12] The EuroQol Group. (2015). EQ-5D-3L User Guide. Basic information on how to use the EQ-5D-3L instrument. https://euroqol.org/publications/user-guides/.

[13] Greiner W, Weijnen T, Nieuwenhuizen M, Oppe S, Badia X, Busschbach J (2003). A single European currency for EQ-5D health states: Results from a six-country study. The European Journal of Health Economics.

[14] Szende, A., Devlin, N., & M., O. (2018). EQ-5D index calculator. https://www.economicsnetwork.ac.uk/health/Other_resources.

[15] Ankri J, Beaufils B, Novella JL, Morrone I, Guillemin F, Jolly D (2003). Use of the EQ-5D among patients suffering from dementia. Journal of Clinical Epidemiology.

[16] Aguirre E, Kang S, Hoare Z, Edwards RT, Orrell M (2016). How does the EQ-5D perform when measuring quality of life in dementia against two other dementia-specific outcome measures?. Quality of Life Research.

[17] Wolfs CA, Dirksen CD, Kessels A, Willems DC, Verhey FR, Severens JL (2007). Performance of the EQ-5D and the EQ-5D+C in elderly patients with cognitive impairments. Health and Quality of Life Outcomes.

[18] Alagic V, Staudinger B (2011). Quality of life in German nursing homes—Results of a survey using the EQ-5D questionnaire. Gesundheitswesen.

[19] Budnick A, Kuhnert R, Konner F, Kalinowski S, Kreutz R, Drager D (2016). Validation of a modified german version of the brief pain inventory for use in nursing home residents with chronic pain. The Journal of Pain.

[20] Yesavage JA, Brink TL, Rose TL, Lum O, Huang V, Adey M (1983). Development and validation of a Geriatric Depression Screening Scale—A preliminary-report. Journal of Psychiatric Research.

[21] Allgaier AK, Kramer D, Mergl R, Fejtkova S, Hegerl U (2011). Validity of the Geriatric Depression Scale in nursing home residents: Comparison of GDS-15, GDS-8, and GDS-4. Psychiatrische Praxis.

[22] Menne HL, Judge KS, Whitlatch CJ (2009). Predictors of quality of life for individuals with dementia. Dementia (London).

[23] Barbe C, Jolly D, Morrone I, Wolak-Thierry A, Drame M, Novella JL (2018). Factors associated with quality of life in patients with Alzheimer’s disease. BMC Geriatrics.

[24] Cohen J (1988). Statistical power analysis for the behavioral sciences.

[25] Sosnowski R, Kulpa M, Zietalewicz U, Wolski JK, Nowakowski R, Bakula R (2017). Basic issues concerning health-related quality of life. Central European Journal of Urology.

